# A phase 1b study of transforming growth factor-beta receptor I inhibitor galunisertib in combination with sorafenib in Japanese patients with unresectable hepatocellular carcinoma

**DOI:** 10.1007/s10637-018-0636-3

**Published:** 2018-07-11

**Authors:** Masafumi Ikeda, Manabu Morimoto, Masaomi Tajimi, Koichi Inoue, Karim A. Benhadji, Michael M. F. Lahn, Daisuke Sakai

**Affiliations:** 1 National Cancer Center Hospital East, 6-5-1 Kashiwanoha, Kashiwa-shi, Chiba-ken 277-8577 Japan; 2 Kanagawa Cancer Center, Yokohama, Japan; 3 Eli Lilly Japan K.K, Kobe, Japan; 4 Eli Lilly and Company, New York, NY USA; 5 Formerly Eli Lilly and Company, Indianapolis, IN USA; 6 Osaka University Hospital, Osaka, Japan

**Keywords:** Galunisertib, Hepatocellular carcinoma, Japan, Phase I clinical trial, Sorafenib

## Abstract

*Background* Galunisertib inhibits type I transforming growth factor-beta receptor serine/threonine kinase. The primary objective of this study was to evaluate the safety and tolerability of galunisertib in combination with sorafenib in Japanese patients with unresectable hepatocellular carcinoma. *Patients and methods* This open-label, dose-escalation, multicenter, nonrandomized phase 1b study consisted of two dose levels of galunisertib, 160 or 300 mg/day, in combination with sorafenib 800 mg/day. Galunisertib 80 mg or 150 mg was administered orally twice daily for 14 days followed by 14 days of rest plus sorafenib 400 mg administered orally twice daily for 28 days. The dose-limiting toxicity evaluation was 28 days after the first dose. Safety measures, pharmacokinetics, and antitumor activity were assessed. *Results* Fourteen patients, 7 at each galunisertib dose, were enrolled and treated. Three dose-limiting toxicities were reported for 2 patients. The most common treatment-emergent adverse events (TEAEs) were hypophosphatemia (14 patients [100%]), palmar-plantar erythrodysesthesia syndrome (12 patients [85.7%]), and decreased platelet count (10 patients [71.4%]). The most common grade ≥ 3 TEAEs were hypophosphatemia (10 patients [71.4%]) and palmar-plantar erythrodysesthesia syndrome (7 patients [50.0%]). No grade 5 TEAEs were reported. The pharmacokinetic profile of galunisertib in combination with sorafenib was similar to that previously reported for galunisertib. Eleven patients had a best overall response of stable disease, and 1 patient achieved a partial response by hepatocellular carcinoma-specific modified RECIST. *Conclusions* These data are consistent with the known safety profile for galunisertib and sorafenib and confirm tolerability of the recommended dose of galunisertib (150 mg twice daily for 14 days) in combination with sorafenib in Japanese patients with unresectable hepatocellular carcinoma.

## Introduction

Liver cancer is the fifth most common cancer in men, the ninth most common cancer in women, and the second-leading cause of cancer deaths for both genders worldwide [1]. The incidence of liver cancer varies by geographical region, with the highest reported incidence in East Asia, including Japan [1]. Prognosis for liver cancer also varies between regions. For instance, an Asia-Pacific study of sorafenib versus placebo in East Asian patients with hepatocellular carcinoma (HCC), which is the most common form of liver cancer [2], showed that median overall survival (OS) in both treatment groups was shorter than the median OS in either group of the global SHARP study of sorafenib versus placebo in patients with HCC [3, 4]. In addition, the GIDEON observational study of sorafenib treatment in patients with HCC found that median time from initial diagnosis to death was 79.6 months in Japan compared to 20.9 months in the Asia-Pacific region, 25.0 months in Europe, 19.5 months in Latin America, and 14.8 months in the United States [5]. Regardless of regional differences, prognosis for liver cancer remains dismal, with the global SHARP study showing a median OS of only 10.7 months for sorafenib and 7.9 months for placebo [4]. Improved therapies for HCC are therefore needed.

Preclinical studies indicate that inhibition of transforming growth factor-beta (TGF-β) may inhibit tumor cell invasion and metastasis, reduce tumor neoangiogenesis, and enhance antitumor immunity [6–8]. Galunisertib is a small molecule designed to selectively inhibit the serine/threonine kinase of the TGF-β receptor type I. Clinical studies have shown favorable short- and long-term toxicity profiles and efficacy with a galunisertib dose range of 40 mg to 300 mg/day in patients with gliomas or advanced cancer [9, 10]. In Japanese patients with advanced solid tumors, galunisertib monotherapy has been shown to have an acceptable tolerability and safety profile with no dose-limiting toxicities (DLTs) [11]. Furthermore, galunisertib in combination with gemcitabine has been shown to have an acceptable tolerability and safety profile with evidence of antitumor activity in Japanese patients with metastatic or locally advanced pancreatic cancer [12]. In a phase 2 study in non-Asian countries, in patients with advanced HCC who had progressed on sorafenib or were ineligible to receive sorafenib, galunisertib monotherapy was shown to have an acceptable safety profile without cardiotoxicity [13]. The median OS was 7.2 months (90% confidence interval [CI], 5.3–9.3) in patients with baseline alpha-fetoprotein (AFP) ≥1.5 ULN (part A) and 16.8 months (90% CI, 10.4–24.1) in patients with AFP <1.5 ULN (part B), with a median time-to-progression (TTP) of 2.7 months (90% CI, 1.4−2.9) in part A and 4.1 months (90% CI, 2.3–5.5) in part B [13]. Collectively, these studies support the evaluation of a combination regimen of a well-tolerated agent such as galunisertib with an existing therapy such as sorafenib in patients with HCC.

The purpose of this phase 1b study was to evaluate the safety and tolerability of galunisertib in combination with sorafenib in Japanese patients with HCC.

## Patients and methods

### Eligibility

Japanese patients at least 20 years of age with HCC not amenable to curative therapy, who were Child-Pugh Class A, who had not received sorafenib previously, and who had an Eastern Cooperative Oncology Group (ECOG) performance status of 0 or 1 and adequate organ function were enrolled. Patients must have had the presence of a measurable or evaluable lesion per Response Evaluation Criteria in Solid Tumors (RECIST) v1.1 and had resolution of all toxic effects of prior therapies before enrollment. Exclusion criteria included major surgery, hepatic locoregional therapy, or systemic chemotherapy within 28 days before enrollment, serious pre-existing medical condition(s), active infection(s), or a previous or concurrent malignancy.

### Study design and treatment

All eligible patients in this phase 1b, open-label, dose-escalation, multicenter, nonrandomized study received oral administration of galunisertib (160 mg or 300 mg/day) in combination with oral administration of sorafenib 800 mg/day. A cycle was defined as 28 days. Galunisertib was administered at either 80 mg twice daily or 150 mg twice daily for 14 days, followed by 14 days of galunisertib treatment rest, and sorafenib 400 mg twice daily for 28 days. Patients continued treatment until a discontinuation criterion was met.

The DLT evaluation period was 28 days after the first dose. Tolerability was defined as a dose where 2 or fewer of 6 patients experience DLTs. A minimum of 3 patients were planned to be enrolled in the galunisertib 160 mg/day plus sorafenib group, and a minimum of 6 patients were planned to be enrolled in the galunisertib 300 mg/day plus sorafenib group. Dose escalation to galunisertib 300 mg/day could occur after confirmation by the sponsor of DLT assessments provided by investigators.

The primary objective of this study was to evaluate the safety and tolerability of galunisertib when combined with sorafenib. The secondary objectives were to characterize the pharmacokinetic (PK) profile of galunisertib and sorafenib and evaluate TTP, progression-free survival (PFS), and antitumor response per RECIST version 1.1 as well as the HCC-specific modified RECIST (mRECIST) [14]. Clinicaltrials.gov identifier: NCT02240433.

### Safety evaluations

Safety analyses included the extent of exposure, adverse events, DLTs, clinical laboratory tests, cardiac monitoring, and other safety evaluations, such as vital signs and ECOG performance status. Adverse events were listed and summarized using the Common Terminology Criteria for Adverse Events (CTCAE version 4.03) and the Medical Dictionary for Regulatory Activities version 20.0.

A DLT was defined as an adverse event during cycle 1 in each treatment group that was possibly related to galunisertib, or the combination of galunisertib plus sorafenib, and fulfilled any of the following criteria using the CTCAE version 4.03: 1) ≥ grade 3 nonhematologic toxicity, except alopecia, fatigue, skin rash, nausea, vomiting, constipation, diarrhea, or electrolyte disturbance, that can be controlled with treatment or optimal supportive care; transient (≤7 days) elevations of alanine aminotransferase, aspartate aminotransferase, alkaline phosphatase, and/or gamma glutamyltransferase; hand-foot syndrome attributed to sorafenib only; or hypertension attributed to sorafenib that cannot be adequately controlled, 2) hematological toxicity, 3) any other toxicity deemed to be dose limiting, or 4) failure to recover sufficiently from toxicities to allow restarting galunisertib after stopping administration due to toxicities.

QT analyses were performed with Bazett and Fridericia correction; corrected QT interval (QTc) was summarized by actual value.

### Pharmacokinetic analyses

PK analyses were conducted on patients who received at least one dose of galunisertib and had PK samples collected. The PK parameters for galunisertib were calculated by standard noncompartmental methods of analysis using Phoenix™ WinNonlin® 6.4 (Pharsight, A Certara Company; Princeton, NJ, USA). The maximum plasma concentration, time to maximum plasma concentration (t_max_), area under the plasma concentration versus time curve from time zero to the last time point with a measurable concentration, and area under the plasma concentration-time curve from time zero to infinity, half-life (t_1/2_), apparent total body clearance (CL/F), and apparent volume of distribution during the terminal phase (V_z_/F) that could be calculated from the data were reported for galunisertib from the noncompartmental PK analyses. Sorafenib PK profiles on day 22 (sorafenib monotherapy) were compared with sorafenib PK profiles on day 14 (administration of galunisertib in combination with sorafenib) within the same patient and in each cohort.

### Efficacy evaluations

Tumor responses were evaluated by RECIST version 1.1 as well as by mRECIST. A complete response (CR) or partial response (PR) was claimed only if the criteria for each were met at a subsequent time point at least 4 weeks later. Duration of response was defined as the start of achieving a response (the first observation of response before confirmation) to the time of disease progression. Duration of response was assessed for responders who achieved a CR or PR. Stable disease (SD) criteria had to be met at least once and at least 6 weeks after the first dose of study drug. TTP and PFS were assessed for all evaluable patients by RECIST and the HCC-specific mRECIST. Levels of AFP were assessed by a central laboratory until data cut-off and then locally according to local procedures after data cut-off.

### Statistical analyses

The sample size was determined based on the study design to evaluate the DLTs, safety, and tolerability of galunisertib in two sequential study treatment groups. Patients who had at least one DLT were evaluable for DLTs. Patients without a DLT who received <80% of the galunisertib dose in cycle 1 for reasons other than drug-related toxicity were considered nonevaluable for DLTs and replaced by a new patient. Patients without a DLT who discontinued the study in cycle 1 due to disease progression, or discontinued sorafenib in cycle 1 for reasons other than disease progression, were assessed for appropriateness of DLT evaluation per Efficacy and Safety Evaluation Committee recommendations. Other safety and efficacy analyses were performed on all patients who received at least one dose of study drug.

## Results

### Patients

All 14 enrolled patients were Japanese, with a median age of 67.5 years (range 50 to 80 years) (Table 1). Most patients (12 patients [85.7%]) were male, and all patients had a histopathological diagnosis of HCC, a baseline ECOG performance status of 0 or 1, and disease classified as Child-Pugh Stage A. A total of 12 patients (85.7%) had undergone at least one prior anticancer therapy. Prior anticancer therapies included transarterial chemoemobilization (11 patients), surgery of curative intent (7 patients), radiofrequency ablation (3 patients), palliative radiotherapy (2 patients), and percutaneous ethanol injection (1 patient). No patients had received prior systemic anticancer therapies. In the galunisertib 160 mg/day plus sorafenib group, 5 patients had T2 tumor/node/metastasis (TNM) classification, 1 patient had T3b, and 1 patient had T4, and in the galunisertib 300 mg/day plus sorafenib group, 3 patients had T2, 1 patient had T3a, 1 patient had T3b, and 2 patients had Tx. In the galunisertib 160 mg and 300 mg/day groups, 3 patients and 4 patients had M1 TNM classification, respectively. In the galunisertib 160 mg/day plus sorafenib group, 2 patients had baseline AFP >400 μg/L and 5 patients had AFP <15 μg/L, with a maximum baseline AFP level of 4140.8 μg/L. In the galunisertib 300 mg/day plus sorafenib group, 5 patients had baseline AFP <10 μg/L, with a maximum baseline AFP level of 70.1 μg/L.

**Table 1:** Baseline demographic and clinical characteristics

	Galunisertib 160 mg/day plus sorafenib *N* = 7	Galunisertib 300 mg/day plus sorafenib *N* = 7	Total *N* = 14
Age, years			
Median (range)	68 (50–79)	67 (54–80)	68 (50–80)
Gender			
Female	1 (14.3)	1 (14.3)	2 (14.3)
Male	6 (85.7)	6 (85.7)	12 (85.7)
Weight, kg			
Median (range)	62 (44–80)	65 (42–85)	65 (42–85)
Pathological diagnosis			
Hepatocellular carcinoma	7 (100)	7 (100)	14 (100)
Disease etiology			
Alcohol use	1 (14.3)	3 (42.9)	4 (28.6)
Hepatitis B	2 (28.6)	2 (28.6)	4 (28.6)
Hepatitis C	3 (42.9)	2 (28.6)	5 (35.7)
Nonalcoholic fatty liver disease	1 (14.3)	0	1 (7.1)
ECOG PS			
0	6 (85.7)	5 (71.4)	11 (78.6)
1	1 (14.3)	2 (28.6)	3 (21.4)
Child-Pugh Stage A	7 (100)	7 (100)	14 (100)

*Abbreviations: ECOG PS* Eastern Cooperative Oncology Group performance status. Data are *n* (%) unless otherwise indicated

### Safety

DLTs were reported by 2 patients who experienced a total of three DLTs. One patient, who received galunisertib 160 mg/day plus sorafenib, experienced a DLT of grade 3 hepatobiliary disorder (acute hepatitis) in cycle 1 from study days 25 to 70 that was reported as a serious adverse event (SAE) related to both study drugs. This patient also experienced a DLT of grade 3 hypophosphatemia in cycle 1 from study days 28 to 37 that was related to both study drugs. Both toxicities resolved, and the patient discontinued treatment per the study protocol. The other patient, who received galunisertib 300 mg/day plus sorafenib, experienced a DLT of grade 3 erythema multiforme in cycle 1 from study days 12 to 19 that was reported as an SAE related to both study drugs. This toxicity resolved, and the patient discontinued treatment per the study protocol.

All treatment-emergent adverse events (TEAEs) reported in this study, regardless of grade, were considered related to study drug(s) (Table 2). All 14 enrolled patients reported at least one TEAE related to study drug(s). The most common TEAEs of any grade were hypophosphatemia (14 patients [100%]), palmar-plantar erythrodysesthesia syndrome (12 patients [85.7%]), and decreased platelet count (10 patients [71.4%]) (Table 2). A total of 13 patients (92.9%) had grade ≥ 3 TEAEs related to study drug(s). The most common grade ≥ 3 TEAEs were hypophosphatemia (10 patients [71.4%]) and palmar-plantar erythrodysesthesia syndrome (7 patients [50.0%]) (Table 2). Two TEAEs were reported as grade 4, both in the galunisertib 300 mg/day plus sorafenib group; these were increased serum amylase related to sorafenib, which did not require dose adjustment, and hepatobiliary disorder related to both study drugs, which led to study discontinuation. No grade 5 TEAEs or deaths were reported. A total of 11 patients (78.6%) had dose reductions, and 8 patients (57.1%) had a cycle delay.

**Table 2:** Treatment-emergent adverse events related to study treatment

	Galunisertib 160 mg/day plus sorafenib *N* = 7			Galunisertib 300 mg/day plus sorafenib *N* = 7		
	Grade 1–2	Grade ≥ 3	Any grade	Grade 1–2	Grade ≥ 3	Any grade
Hypophosphatemia	1 (14)	6 (86)	7 (100)	3 (43)	4 (57)	7 (100)
Palmar-plantar erythrodysesthesia syndrome	2 (29)	4 (57)	6 (86)	3 (43)	3 (43)	6 (86)
Platelet count decreased	4 (57)	0	4 (57)	4 (57)	2 (29)	6 (86)
Alopecia	3 (43)	0	3 (43)	1 (14)	0	1 (14)
Anorexia	1 (14)	1 (14)	2 (29)	2 (29)	0	2 (29)
Creatinine phosphokinase increased	2 (29)	0	2 (29)	2 (29)	0	2 (29)
Rash maculopapular	0	0	0	4 (57)	0	4 (57)
Aspartate aminotransferase increased	1 (14)	0	1 (14)	2 (29)	0	2 (29)
Diarrhea	2 (29)	0	2 (29)	1 (14)	0	1 (14)
Fatigue	2 (29)	0	2 (29)	1 (14)	0	1 (14)
Fever	2 (29)	0	2 (29)	1 (14)	0	1 (14)
Hoarseness	2 (29)	0	2 (29)	1 (14)	0	1 (14)
Nausea	1 (14)	0	1 (14)	2 (29)	0	2 (29)
Serum amylase increased	0	0	0	1 (14)	2 (29)	3 (43)
Anemia	0	1 (14)	1 (14)	1 (14)	0	1 (14)
Constipation	1 (14)	0	1 (14)	1 (14)	0	1 (14)
Dysgeusia	2 (29)	0	2 (29)	0	0	0
Hepatobiliary disorders	0	1 (14)	1 (14)	0	1 (14)	1 (14)
Hyperglycemia	2 (29)	0	2 (29)	0	0	0
Hypertension	1 (14)	0	1 (14)	0	1 (14)	1 (14)
C-reactive protein increased^a^	0	0	0	2 (29)	0	2 (29)
Mucositis oral	1 (14)	0	1 (14)	1 (14)	0	1 (14)
Neutrophil count decreased	2 (29)	0	2 (29)	0	0	0
Rash acneiform	2 (29)	0	2 (29)	0	0	0
White blood cell decreased	2 (29)	0	2 (29)	0	0	0

Treatment-emergent adverse events related to study treatment of any grade that were reported in at least two patients are listed by National Cancer Institute Common Terminology Criteria for Adverse Events (CTCAE) preferred term in decreasing frequency of any grade

Data are *n* (%)

^a^The CTCAE preferred term for c-reactive protein increased was “investigations”

A total of six SAEs were reported in 5 patients during the study. These SAEs were grade 2 palmar-plantar erythrodysesthesia syndrome related to sorafenib, grade 3 hypophosphatemia related to both study drugs, grade 3 hepatobiliary disorder (acute hepatitis) related to both study drugs, grade 3 erythema multiforme related to both study drugs, grade 4 hepatobiliary disorder related to both study drugs, and grade 3 biliary tract infection (cholangitis) not considered to be related to either study drug.

A total of 7 patients (50.0%) had at least one abnormal QTc that met International Conference on Harmonisation (ICH) criteria; however, there were no clinically important echocardiography findings, treatment-emergent cardiac laboratory abnormalities, or TEAEs categorized as a cardiac disorder. Most abnormal clinical laboratory values reported during the study were low grade. Grade ≥ 3 clinical laboratory values included low serum phosphate, low calcium, low sodium, low blood lymphocytes, high alanine aminotransferase, high aspartate aminotransferase, high alkaline phosphatase, high bilirubin, and high cholesterol.

### Pharmacokinetics

**Fig. 1:**
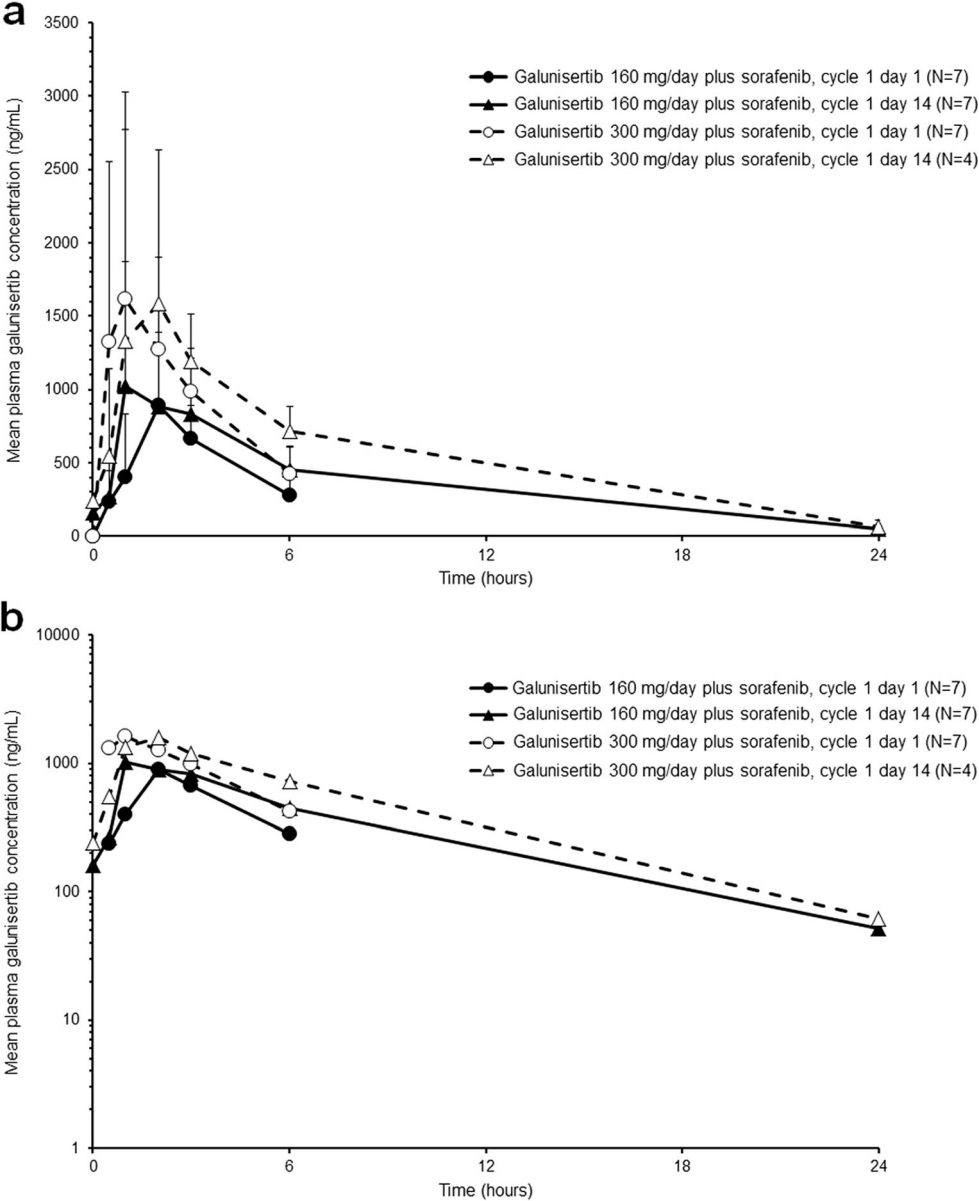
Arithmetic mean (plus standard deviation) plasma galunisertib concentration-time profiles on cycle 1 days 1 and 14 following administration of galunisertib 160 mg/day or 300 mg/day. Linear plot (**a**); semi-logarithmic plot (**b**)

The PK profile of galunisertib was characterized by rapid absorption, with median t_max_ of approximately 1 to 2 h post-dose. Although PK samples for galunisertib were collected only up to 6 h post-dose on cycle 1 day 1, the observed PK of galunisertib was comparable between cycle 1 day 1 and cycle 1 day 14 (Table 3). At steady state, on cycle 1 day 14, the geometric mean t_1/2_ was 4.24 and 4.01 h in the galunisertib 160 mg/day plus sorafenib and galunisertib 300 mg/day plus sorafenib groups, respectively. The geometric mean CL/F and V_z_/F on cycle 1 day 14 were 10.1 L/h and 62.0 L in the galunisertib 160 mg/day plus sorafenib group, and 13.5 L/h and 78.1 L in the galunisertib 300 mg/day plus sorafenib group, respectively (Table 3). These data should be interpreted with caution as they are based on a small number of patients and only 24 h of PK sampling. In addition, PK data in cycle 1 day 14 were not obtained from 3 patients, due to no galunisertib administration on that day. The mean plasma galunisertib concentration-time profiles are shown in Fig. 1. The PK profile of sorafenib in combination with galunisertib was comparable to that of sorafenib administered alone (data not shown).

**Table 3:** Pharmacokinetic parameters of galunisertib

	Galunisertib 160 mg/day		Galunisertib 300 mg/day	
	Cycle 1 Day 1	Cycle 1 Day 14	Cycle 1 Day 1	Cycle 1 Day 14
*N*	7	7	7	4
C_max_ (ng/mL)	899 (37)	1150 (61)	1900 (61)	2000 (74)
t_max_ (h)^a^	1.93 (1.00–3.00)	1.85 (0.92–5.92)	0.93 (0.47–3.25)	2.04 (0.88–5.63)
t_1/2_ (h)^b^	NC	4.24 (3.79–4.86)^d^	NC	4.01 (3.28–4.52)^d^
AUC(0-6) (ng∙h/mL)^c^	2800 (31)	3670 (52)	5040 (49)	5570 (44)
AUC(0–24) (ng∙h/mL)	NC	7170 (21)^e^	NC	10,600 (9)
AUC(0-∞) (ng∙h/mL)	NC	7870 (4)^d^	NC	11,100 (9)^d^
CL/F (L/h)	NC	10.1 (4)^d^	NC	13.5 (9)^d^
V_Z_/F (L)	NC	62.0 (9)^d^	NC	78.1 (10)^d^

Data are geometric mean (CV%) unless otherwise indicated

*Abbreviations: AUC(0-∞)* area under the plasma concentration versus time curve from time zero to infinity, *AUC(0-x)* area under the plasma concentration versus time curve from time zero to x hours after dose, *CL/F* apparent total body clearance, *C*_*max*_ maximum observed plasma concentration, *CV* coefficient of variance, *NC* not calculated, *t*_*1/2*_ half-life associated with the terminal rate constant (lambda z) in noncompartmental analysis, *t*_*max*_ time of maximum observed plasma concentration, *V*_*z*_*/F* apparent volume of distribution during the terminal phase

^a^Median (range)

^b^Geometric mean (range)

^c^AUC(0-t_last_) on cycle 1 days 1 and 14 were reported as AUC(0–6) and AUC(0–24), respectively

^d^*n* = 3

^e^*n* = 6

### Efficacy

**Fig. 2:**
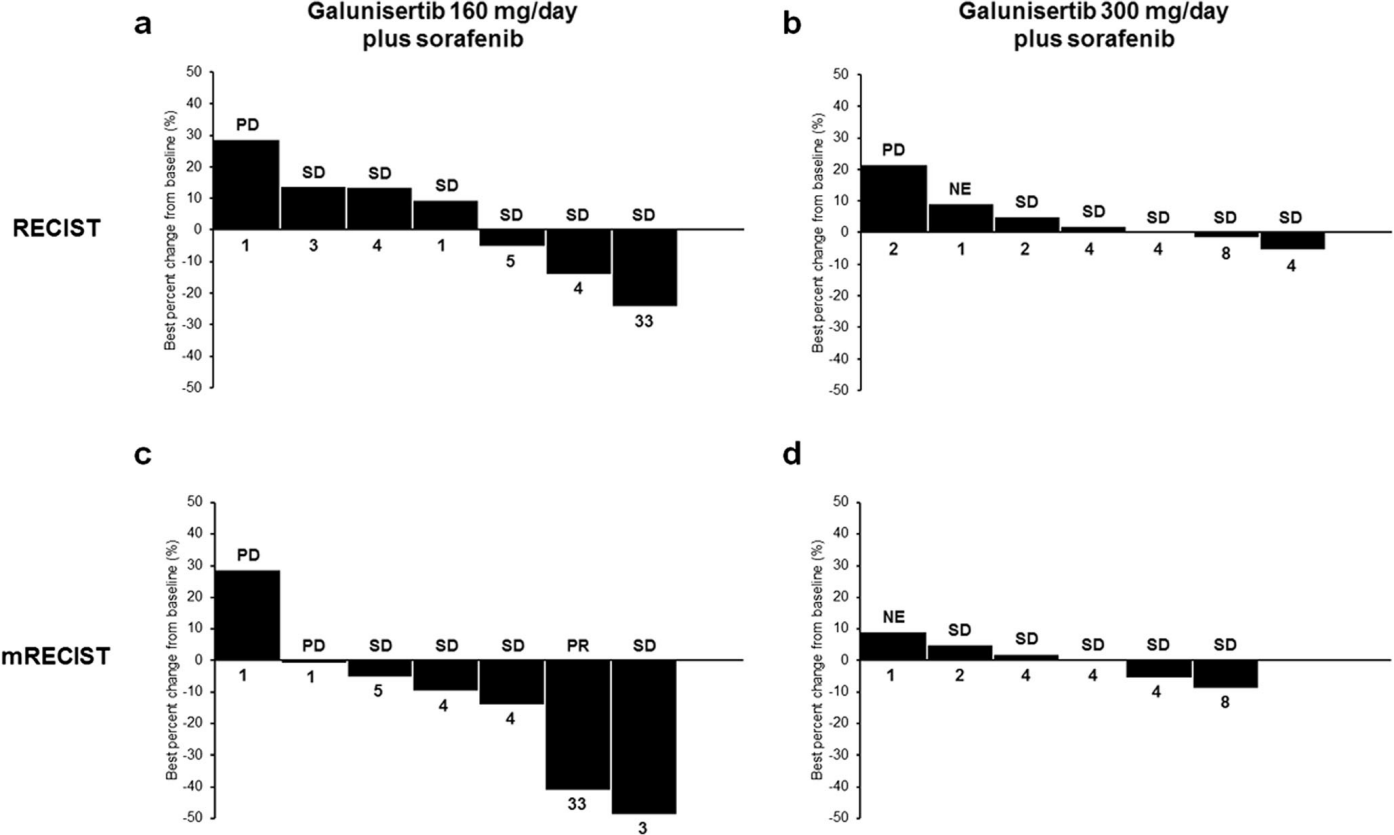
Best percentage change in tumor size with galunisertib 160 mg/day plus sorafenib (**a** and **c**) and galunisertib 300 mg/day plus sorafenib (**b** and **d**). Data are derived from RECIST assessment (**a** and **b**) and mRECIST assessment (**c** and **d**). Numbers below each graph indicate the number of cycles of study treatment received by each patient. Abbreviations: mRECIST = modified Response Evaluation Criteria in Solid Tumors; NE = not evaluable; PD = progressive disease; PR = partial response; RECIST = Response Evaluation Criteria in Solid Tumors; SD = stable disease

The best overall response (BOR) with evaluation by RECIST version 1.1 was SD (6 patients) in the galunisertib 160 mg/day plus sorafenib group, with 1 patient having progressive disease (disease control rate, 85.7%), and SD (5 patients) in the galunisertib 300 mg/day plus sorafenib group, with 1 patient having progressive disease and 1 patient not evaluable (disease control rate, 71.4%). No CR or PR was achieved in either treatment group by RECIST version 1.1 evaluation. The BOR with evaluation by the HCC-specific mRECIST was PR (1 patient) or SD (4 patients) in the galunisertib 160 mg/day plus sorafenib group, with 2 patients having a best response of progressive disease (disease control rate, 71.4%), and SD (5 patients) in the galunisertib 300 mg/day plus sorafenib group, with 1 patient considered not evaluable (disease control rate, 71.4%). Waterfall plots of best percentage change in tumor size of target lesions are shown in Fig. 2.

Both the median TTP and PFS were 3.6 months (range, 1.0 to 30.8+ months) by RECIST version 1.1 evaluation. Both TTP and PFS were generally similar in both treatment groups. One patient in the galunisertib 160 mg/day plus sorafenib group had a longer TTP of 30.8 months after 33 cycles of treatment (censored; still on study) when compared with other patients in the study. This patient was the only one in the study to have a PR and is discussed in further detail below. Analysis by the HCC-specific mRECIST resulted in generally similar TTP and PFS findings to that by RECIST version 1.1.

The levels of AFP did not demonstrate any clear pattern relative to overall tumor response. The patient with a PR by mRECIST evaluation had AFP levels that were either absent/low or initially measurable and then tapered downward (depending on the assay used to detect AFP) during the study. The duration of response for this patient, as assessed by mRECIST, was 29.0 months at time of censorship, at which time this patient was still on study. This patient initiated study treatment with galunisertib 160 mg/day plus sorafenib and had a PR recorded on study day 57 during cycle 3 and an overall PFS of 30.8 months after 33 cycles of treatment.

## Discussion

The data from this phase 1b, open-label, dose-escalation, multicenter, nonrandomized study are consistent with the known safety profiles for galunisertib and sorafenib and confirm tolerability of this combination in Japanese patients with unresectable HCC. A total of three DLTs were experienced by 2 patients; 1 patient with grade 3 hepatobiliary disorder (acute hepatitis) and grade 3 hypophosphatemia, and 1 patient with grade 3 erythema multiforme. The PK profile of galunisertib in combination with sorafenib was similar to that previously reported [9–12]. Seventy-nine percent of patients had a BOR of SD, and assessment using the HCC-specific mRECIST showed 1 patient achieved a PR.

All three DLTs reported in this study were considered related to the drug combination of galunisertib and sorafenib. All DLTs were subsequently reported as resolved and resulted in treatment discontinuation for both patients. Although no definitive DLTs have been reported in previous phase 1 studies [10–12], the number of DLTs reported in this study was considered low and suggests that galunisertib at doses of 80 mg twice daily and 150 mg twice daily, in combination with sorafenib 400 mg twice daily, is safe and tolerable.

The most frequently reported TEAEs were generally balanced across the treatment groups and consistent with the known safety profiles of both galunisertib and sorafenib, including a global study of galunisertib and sorafenib combination therapy in non-Asian patients with advanced HCC [13] and a study of galunisertib monotherapy in Japanese patients with advanced solid tumors [11]. The most frequently reported any-grade TEAE related to galunisertib was hypophosphatemia. The only other galunisertib-related any-grade TEAE reported for more than half of patients overall was decreased platelet count. The most common grade 3/4 TEAEs were hypophosphatemia and palmar-plantar erythrodysesthesia syndrome, the latter of which is typically associated with sorafenib treatment [3]. In contrast to these findings, a global phase 2 study of galunisertib plus sorafenib in patients with advanced HCC reported that the most common grade 3/4 TEAEs were fatigue and embolism [13]. Hypophosphatemia and decreased platelet counts have not been observed in previous galunisertib monotherapy trials, including a study of Caucasians with HCC [10, 11, 13, 15, 16]. Thus, it is unclear whether this safety reporting is partly influenced by the type of patients who received galunisertib in combination with sorafenib in this study. The differences in TEAEs reported in this study compared to previous studies of galunisertib may also be due to the relatively few grade 3/4 TEAEs reported and/or the small patient numbers in this study as well as in previous studies.

Most abnormal low or high laboratory values were low-grade. There was no clear pattern in postbaseline vital signs. All patients maintained their baseline ECOG performance status of 0 or 1 postbaseline, except for 1 patient who discontinued treatment due to an SAE. Half of the patients in the study had at least one abnormal QTc that met ICH criteria, albeit there were no clinically important echocardiography findings, treatment-emergent cardiac laboratory abnormalities, or TEAEs categorized as a cardiac disorder. These findings are consistent with previous studies of galunisertib, which have demonstrated no medically relevant cardiac toxicity [17]. In contrast, vascular endothelial growth factor receptor tyrosine kinase inhibitors, such as sorafenib, have been shown to be associated with QTc prolongation [18, 19]. A total of six SAEs were reported for 5 patients. All but one SAE was related to study drug(s), and all resulted in study drug dose adjustment(s) or discontinuation. Regardless, the SAEs were generally similar across the treatment groups and did not suggest a new safety signal compared to previous studies of galunisertib or sorafenib [3, 9–13, 20].

The galunisertib PK profile was characterized by rapid absorption and elimination at both galunisertib doses (median t_max_ of 1–2 h and mean t_1/2_ of approximately 4 h) and was consistent with the PK profiles in both Japanese and non-Japanese patients with pancreatic cancer [9, 20]. These data should be interpreted with caution as they are based on a small number of patients with only 24 h of PK sampling, and PK data in cycle 1 day 14 were not obtained from 3 patients.

The antitumor activity observed in this study was similar for the 160 mg and 300 mg/day galunisertib doses. The BOR by RECIST version 1.1 evaluation was SD for the majority of patients. Evaluation by HCC-specific mRECIST showed 1 patient in the galunisertib 160 mg/day plus sorafenib group achieved a PR with a stable response of over 30.8 months. Median TTP and PFS were both approximately 3.6 months in this study, regardless of assessment criteria used. This is consistent with the median TTP of 2.7 months (part A) and 4.1 months (part B) reported in the phase 2 global study of galunisertib and sorafenib combination therapy in patients with advanced HCC [13]. Despite the small sample size, the antitumor activity data in this study are promising and suggest galunisertib in combination with sorafenib may be associated with some clinical response in Japanese patients with unresectable HCC.

The main limitation of this study was the small sample size, which is an inherent limitation of the phase 1 design of the study. Furthermore, a conclusive assignment of specific drug association with TEAEs was not possible because this was not a randomized study. The main strength of this study was the enrollment of a single racial population.

In conclusion, the overall safety findings were consistent with the known safety profiles for galunisertib and sorafenib, and confirm tolerability of the recommended dose of galunisertib (150 mg twice daily for 14 days) in combination with sorafenib in Japanese patients with unresectable HCC. The PK profile of galunisertib was similar to that reported previously for Japanese and non-Japanese patients. Galunisertib in combination with sorafenib may be associated with some efficacy. The combination of galunisertib 150 mg twice daily and sorafenib is being investigated in a phase 2 study (ClinicalTrials.gov Identifier: NCT01246986).
